# Omics-Guided Construction of Microbial Consortia for Reproducible Traditional Fermented Foods and Beverages

**DOI:** 10.3390/foods15101643

**Published:** 2026-05-08

**Authors:** Dandan Song, Liang Yang, Chunlin Zhang

**Affiliations:** 1 School of Brewing Engineering, Moutai Institute, Renhuai 564501, China; songdd0330@163.com (D.S.); zcl818075@163.com (C.Z.); 2 Guizhou Key Laboratory of Microbial Resources Exploration in Fermentation Industry, Kweichow Moutai Group, Zunyi 564501, China; 3 Modern Baijiu Brewing Technology Engineering Research Center of Guizhou Universities, Moutai Institute, Renhuai 564501, China; 4 Guizhou Province Technology Innovation Center for Jiangxiangxing Baijiu, Moutai Institute, Renhuai 564501, China

**Keywords:** traditional fermented foods and beverages, defined microbial consortia, multi-omics integration, microbial interactions, reproducible fermentation, synthetic microbial consortia

## Abstract

Traditional fermented foods and beverages (TFFB) rely on complex microbial communities that generate distinctive flavors, nutritional attributes, and cultural value, but spontaneous or empirically controlled fermentations often limit reproducibility. Defined microbial consortia (DMCs) provide a promising route for improving fermentation controllability and product consistency, although overly simplified starters may fail to reproduce the ecological robustness and sensory complexity of traditional systems. This review focuses on how multi-omics and culturomics can support rational DMC design in TFFB. We summarize how metagenomics, metatranscriptomics, metaproteomics, metabolomics, and culturomics reveal community structure, functional potential, active expression, metabolic output, and cultivable strain resources. Particular attention is given to translating multi-omics evidence into strain prioritization through the identification of keystone microorganisms that drive core fermentation functions and helper microorganisms that support ecological or metabolic stability. We further propose an Assembly-Assessment-Redesign (A-A-R) framework for iterative DMC optimization, linking strain selection, functional validation, performance evaluation, and consortium redesign. Finally, we discuss key challenges, including cross-omics integration, experimental verification of microbial functions, standardized validation criteria, and the transfer of laboratory-designed consortia to industrial fermentation systems.

## 1. Introduction

Fermentation is an ancient technology used to extend food shelf life and/or enhance flavor [1]. Traditional fermented foods and beverages (TFFB) constitute important components of daily diets in many countries and regions, encompassing alcoholic beverages, dairy products, soybean products, cereal-based foods, fermented vegetables, and a variety of region-specific drinks. Although recent studies have greatly advanced the understanding of fermented food microbiomes through omics technologies, many reviews still focus mainly on microbial diversity, single omics applications, or general starter-culture development. A clear gap remains between describing complex microbial communities and converting such information into practical rules for defined microbial consortia (DMC) design. In particular, how to identify functionally important members, distinguish keystone and helper microorganisms, validate their roles, and redesign microbial combinations for reproducible fermentation has not yet been sufficiently clarified. Therefore, this review aims to synthesize multi-omics evidence from traditional fermentation systems and develop a more design-oriented perspective for DMC construction. Its originality lies in connecting microbial detection, functional interpretation, strain validation, and iterative consortium refinement within a unified framework, rather than treating omics tools as separate descriptive methods.

**Figure 1:**
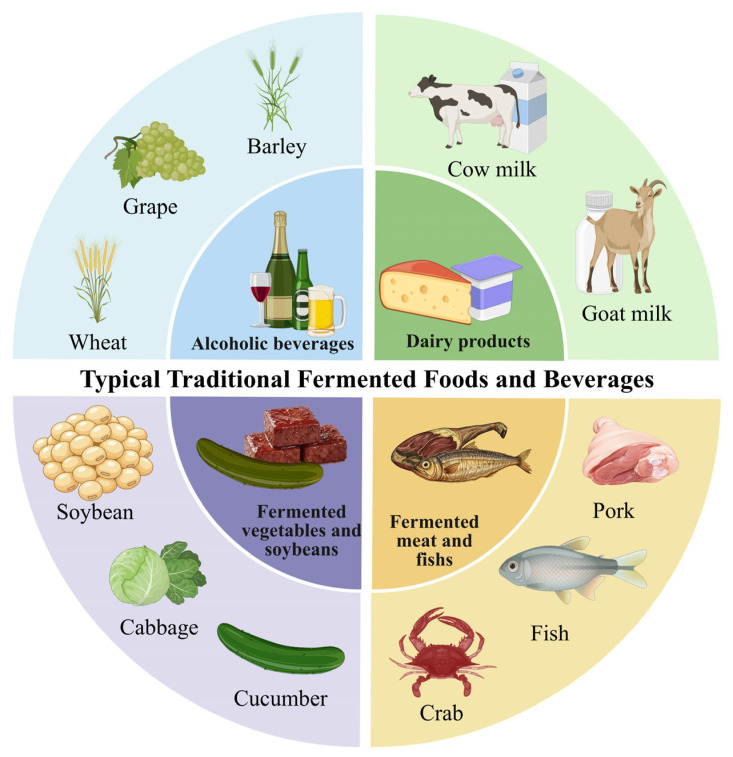
Types of main Fermented foods and beverages.

Representative examples include Chinese Baijiu and douchi, Japanese sake, French wine, Korean cheonggukjang, Indonesian tempeh, Japanese natto and miso, traditional Italian vinegar, Spanish sherry, and European dark beer (Figure 1) [2,3]. These products are highly appreciated by consumers and possess considerable dietary and social value. Their significance lies not only in improvements in flavor, texture, nutrition, and preservation, but also in the artisanal knowledge, microbial resources, and cultural heritage they embody [4,5]. In this review, defined microbial consortia (DMCs) refer to intentionally selected, compositionally known, and reproducibly assembled microbial communities designed to perform specific fermentation functions under controlled conditions [6,7,8]. The keystone microbiome is defined here as a group of microorganisms that exert disproportionately important effects on community structure, metabolic function, fermentation stability, or product quality, even when their relative abundance is not dominant [9,10,11]. By contrast, the helper microbiome refers to microorganisms that may not directly determine the core fermentation phenotype but support DMC performance through complementary functions, such as nutrient exchange, stress tolerance, cross-feeding, flavor modulation, or ecological stabilization [6,12]. These definitions provide the conceptual basis for linking traditional fermentation ecosystems with rational consortium design.

Compared with highly standardized industrial foods, traditional fermentation systems usually rely on the combined action of indigenous raw-material microbiota, utensil- and environment-associated microorganisms, and complex inoculation systems such as back-slopping, recycled fermented grains, aged pits, Qu, a traditional Chinese fermentation starter prepared from cereal- or bran-based substrates and enriched with mixed molds, yeasts, and bacteria, or active dry yeast [1,2,13,14]. Consequently, they are essentially spontaneous, multispecies fermentation ecosystems characterized by stage-specific succession and strong environmental regulation [1]. To better understand TFFB, numerous studies have investigated fermentation processes [15], the isolation and identification of representative microorganisms [16,17,18], microbial diversity [19], flavor composition [20], nutritional value [21], safety indicators [22,23], and resource utilization [24,25,26,27]. Collectively, these studies indicate that although spontaneous or non-strictly defined starter-dominated systems can generate rich and complex flavors, their community composition, functional expression, and metabolic outputs often vary across batches, seasons, regions, and process microenvironments. As a result, product quality remains highly dependent on empirical control, which limits reproducibility under industrial conditions [28,29].

One of the central challenges currently facing TFFB is the imbalance between the formation of distinctive flavors and the achievement of stable, controllable production. On the one hand, bacteria, yeasts, and molds in spontaneous fermentation systems collectively generate characteristic aromas and tastes through carbohydrate degradation, protein hydrolysis, lipid transformation, and secondary metabolism [5]. On the other hand, because these systems often operate under open or semi-open conditions, factors such as raw-material quality, environmental temperature and humidity, ventilation and oxygen availability, biofilms on equipment surfaces, inoculation history, and hygiene control can all influence microbial community assembly and succession. These influences ultimately lead to marked fluctuations in fermentation rate, product composition, flavor profile, and sensory quality across batches [28,29].

To address the instability and inconsistency arising from reliance on undefined microbial communities, modern food fermentation industries have long tended to adopt defined starters or commercial starter cultures in order to improve process controllability, reduce fermentation failure, and enhance product standardization. Indeed, studies have shown that the use of defined starters helps achieve more stable sensory characteristics and more consistent product quality. In this context, the concept of defined microbial consortia (DMCs) has emerged. DMCs are reconstructed communities whose microbial composition is defined and/or deliberately assembled to produce specific metabolites, flavor compounds, or other desired end products [30]. However, for many TFFB, simplified starters composed of a single strain or only a few strains often fail to fully reproduce the extensive metabolic division of labor, cross-kingdom interactions, nutritional cross-feeding, and spatiotemporal succession that occur in natural fermentation ecosystems. Consequently, they are often unable to preserve the complex flavor profiles and ecological robustness that characterize traditional products [31].

Moreover, the design and construction of DMCs themselves face several major challenges. The first is the difficulty of obtaining target strains. Although many functional strains have already been isolated from fermentation environments, a large proportion of microorganisms remain uncultured or difficult to culture. Therefore, there is a need not only to improve strain-isolation technologies, but also to acquire reliable taxonomic and genetic information for target strains. The second challenge concerns the strategies used to construct synthetic microbial communities. At present, synthetic consortia are mainly designed through top-down and bottom-up approaches [8]. The former seeks to simplify naturally occurring microbial communities into representative consortia, whereas the latter focuses on deliberately constructing new communities based on explicit goals or design principles [7]. Both approaches rely on a comprehensive understanding of the roles that microorganisms play in driving key biochemical transformations and influencing the quality of traditional fermented foods, as well as on genome-informed knowledge of community composition and metabolic potential [32]. Nevertheless, regardless of whether a top-down or bottom-up strategy is used, reconstructed communities still face a critical limitation when participating in or dominating fermentation: the resulting products may partially lose the regional typicity and flavor complexity characteristic of TFFB, manifesting as reduced flavor intensity, simplified sensory layers, or even the absence of certain key bioactive compounds.

The development of multi-omics technologies provides an important route for overcoming this bottleneck. Current studies indicate that metagenomics, metatranscriptomics, metaproteomics, metabolomics, and culturomics can characterize fermentation microecology from the perspectives of community composition, functional potential, active expression, terminal metabolites, and cultivable strain resources, respectively. More importantly, integrated multi-omics approaches can capture the microbial–molecular complexity of fermentation systems more effectively than any single omics technique, thereby enabling deeper insights into the intrinsic mechanisms underlying flavor formation, quality development, and the accumulation of functional products [33,34,35]. Such a systematic framework, spanning community structure, community function, metabolic phenotype, and product quality, not only provides a theoretical basis for reproducible and controllable traditional fermentation, but also offers methodological support for shifting microbial consortium design and optimization from an empiricism-driven mode to a mechanism-driven one. In this way, research on TFFB is gradually moving beyond a “black-box” empirical process toward a complex biological system that can be hierarchically observed, quantitatively associated, and rationally regulated.

Although advances in omics technologies and bioinformatics have greatly promoted research on reproducible fermentation, the application of multi-omics remains limited in several respects. First, criteria for screening and evaluating core functional microorganisms have not yet been standardized. Second, systems for community assembly, interaction validation, and stability assessment remain insufficiently developed. Third, the construction of functionally stable and controllable synthetic consortia is still challenging. Fourth, scalable application pathways oriented toward target traits and safety are still unclear. To address these issues, this narrative review focused on peer-reviewed studies related to traditional fermented foods, multi-omics technologies, microbial interaction analysis, DMC construction, synthetic or defined microbial communities, and fermentation quality regulation. The literature was selected by considering its relevance to fermented food ecosystems, its methodological connection with omics-guided microbial identification or functional validation, and its contribution to DMC assembly, assessment, or application. Recent studies were prioritized, while representative conceptual and methodological papers were also included when they were necessary for defining key terms or explaining the development of the field. Accordingly, this review summarizes how multi-omics can facilitate the construction of synthetic microbial consortia, then reviews recent advances in the application of DMCs in fermented food production, and finally proposes a standardized workflow for DMC construction and implementation. Compared with recent reviews that mainly summarize individual omics techniques, microbial succession, or synthetic consortium applications, this review places greater emphasis on the translation of multi-omics evidence into DMC design logic. Specifically, it highlights the keystone/helper microbiome concept as a functional screening strategy and the Assembly-Assessment-Redesign (A-A-R) framework as an iterative workflow connecting strain selection, consortium validation, performance evaluation, and redesign. This perspective aims to clarify how descriptive omics data can be transformed into experimentally testable and practically applicable fermentation consortia.

This review was developed through a narrative literature search combined with a brief bibliometric overview. The literature was searched in Web of Science Core Collection, Scopus, PubMed, ScienceDirect, and Google Scholar. The search covered studies published from 1998 to 2026. Search terms included traditional fermented foods, fermented beverages, multiomics, metagenomics, metatranscriptomics, metaproteomics, metabolomics, culturomics, defined microbial consortia, synthetic microbial consortia, keystone microbiome, helper microbiome, microbial interaction, and fermentation quality. Articles were included when they addressed traditional fermentation systems, omics based microbial analysis, functional strain validation, DMC construction, or fermentation quality regulation. Studies were excluded when they were unrelated to food fermentation, lacked clear microbial or omics information, or were not peer reviewed. This strategy was used to identify both recent progress and representative studies, while reducing over reliance on a single food system.

## 2. Omics Analyses of Great Relevance to Fermented Food Analysis and Reconstruction

### 2.1. Metagenomics

**Figure 2:**
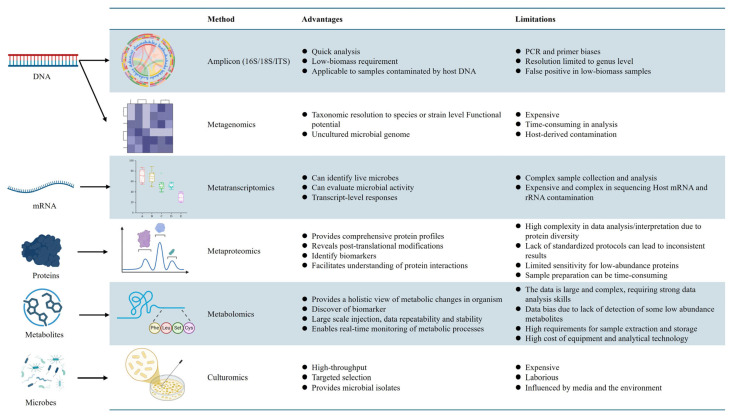
Advantages and limitations of high-throughput phenotyping methods used in microbiome research.

Metagenomics was first established in 1998 and aims to elucidate the genomes of multiple microbial species by directly extracting nucleic acids from complex biological samples without cultivation [36,37]. In fermented food studies, metagenomic strategies are mainly divided into amplicon sequencing and shotgun metagenomics. Amplicon sequencing targets marker genes, such as 16S rRNA for prokaryotes and 18S rRNA or ITS regions for eukaryotes, and is mainly used to compare microbial community structures across fermentation stages, regions, or processing conditions (Figure 2) [33,38]. Shotgun metagenomics sequences total community DNA and can provide higher-resolution taxonomic information and functional gene profiles [33,38].

**Figure 3:**
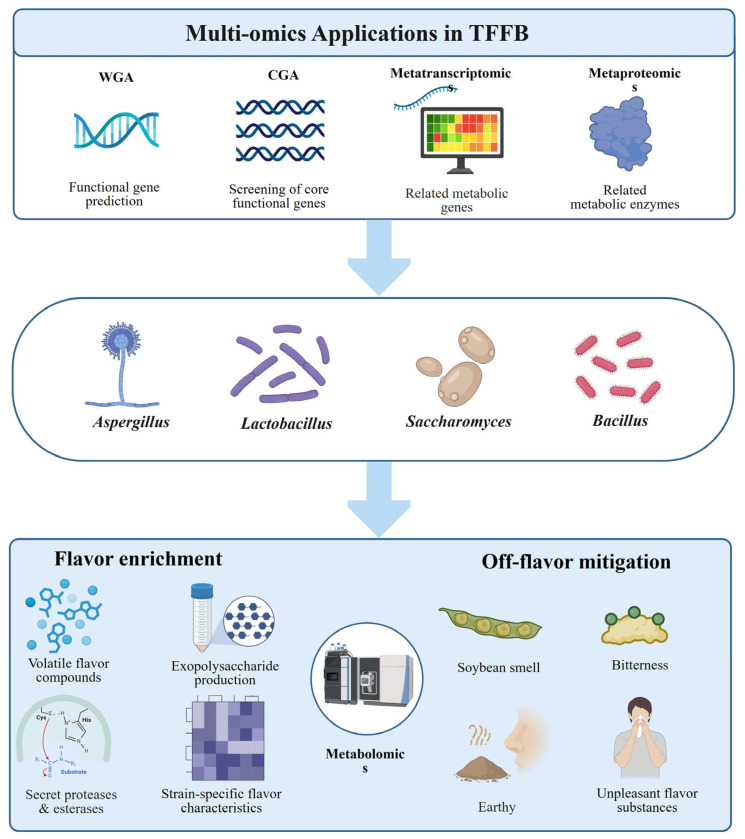
Multi-omics strategies used to facilitate the establishment of microbial consortia by providing multi-perspective information. Note: Abbreviations: TFFB, traditional fermented foods and beverages; WGA, whole genome analysis; CGA, comparative genomic analysis; Cys, cysteine; His, histidine.

In fermented food research, metagenomics is valuable because it reveals microbial succession, core taxa, and functional potential without relying on microbial isolation (Figure 3) [39]. Amplicon sequencing can rapidly screen microbial differences related to flavor formation, product stability, and safety [40], whereas shotgun metagenomics can further identify genes and pathways involved in carbohydrate metabolism, amino acid conversion, organic acid production, lipid transformation, and volatile flavor compound biosynthesis [41]. This approach has been applied to alcoholic beverages, fermented meat products, fermented vegetables, dairy products, and fermented soybean products for community profiling, functional microorganism tracing, flavor-network prediction, and risk-related gene screening, such as biogenic amine formation [42]. When combined with metagenome assembly, metagenome-assembled genome reconstruction, and functional annotation, metagenomics can link microbial taxa with metabolic potential and quality-related phenotypes, thereby supporting fermentation regulation, functional strain screening, and synthetic consortium design [43].

Benefiting from the rapid development of metagenomics, many studies have reported important findings on the microbiomes and metabolic potential of TFFB. For example, analysis of Baijiu fermented grains sampled along the riverbank from upstream to downstream showed that *Acetobacter pasteurianus*, *Saccharomyces cerevisiae*, *Wickerhamomyces anomalus*, and *Pichia* spp. constituted the core microbiome of Baijiu fermented grains [44]. Meanwhile, the core microbiome of Baijiu fermentation systems has also been systematically summarized and mainly includes lactic acid bacteria such as *Limosilactobacillus panis*, *Lactiplantibacillus plantarum*, and *Limosilactobacillus fermentum*; bacilli such as *Bacillus amyloliquefaciens* and *Bacillus subtilis*; as well as *Weissella* spp., *Sphingobacterium* spp., *Enterobacter* spp., and *Kroppenstedtia eburnean* [45,46,47,48]. Core yeast taxa mainly include *Saccharomyces cerevisiae*, *Schizosaccharomyces pombe*, *Pichia kudriavzevii*, *Candida inconspicua*, and *Candida castellii*, as well as *Pichia* spp., *Torulaspora* spp., *Saccharomyces* spp., *Candida* spp., *Schizosaccharomyces* spp., and *Zygosaccharomyces* spp. [49,50]. In addition, filamentous fungi such as *Rhizopus* and *Aspergillus* are also regarded as important core microbial groups during Baijiu fermentation [51]. In another study examining changes in the grape berry surface microbiome during grape development, grapes were found to recruit microorganisms according to developmental stage while still retaining a core microbiome. A relatively stable core microbiome was present on the grape surface, mainly consisting of fermentation-related yeasts, yeast-like fungi, filamentous fungi, and several bacterial taxa closely associated with alcoholic fermentation. Among them, genera such as *Saccharomyces*, *Candida*, *Aureobasidium*, *Cladosporium*, *Alternaria*, *Fusarium*, *Penicillium*, *Leuconostoc*, *LAB*, and *Gluconobacter* were repeatedly identified as core members [52]. These core microorganisms persisted across different production regions and developmental stages, although their relative abundances changed with fruit development, suggesting that they may play important roles in grape maturation, microbial colonization, and the establishment of subsequent fermentation characteristics.

Although considerable evidence has accumulated demonstrating the value of metagenomics in revealing microbial composition and functional potential in traditional fermented food and beverage systems, these studies still have important limitations in accurately detecting and describing in situ microbial activity. In other words, microorganisms detected by metagenomics may include dead cells, and cell activity cannot be directly inferred. In addition, amplicon sequencing is easily influenced by primer choice, PCR amplification bias, and differences in marker gene copy number, which may lead to the over- or underestimation of certain taxa and limit its resolution at the strain level (Figure 2) [36,53]. Although shotgun metagenomics provides higher resolution, it still faces challenges such as host DNA interference, high sequencing depth requirements, and relatively high analytical costs. Moreover, it still encounters major bottlenecks in assembly and annotation when applied to complex fermentation systems [54]. Because microbial communities often contain members with substantial abundance differences as well as highly similar closely related strains, short-read datasets frequently fail to achieve complete assemblies and may generate fragmented sequences, breaks in repetitive regions, and chimeric assemblies involving genomes from different strains, which further affect the completeness and accuracy of MAGs [42]. In terms of downstream analysis, even when relatively high-quality assemblies are obtained, functional annotation still depends heavily on existing databases. However, a considerable proportion of uncultured or insufficiently studied microorganisms in fermented foods still lack adequate reference genomes, which limits the accuracy of functional gene identification and metabolic pathway inference.

Furthermore, the interpretation of metagenomic data is influenced by a variety of experimental and biological factors. Parameters such as sequencing depth, sampling strategy, and the choice between replicate sampling within the same product and comparative sampling across different fermented foods can all affect the apparent diversity and structure of microbial communities [55,56,57]. In addition, because substrate composition, processing conditions, and fermentation dynamics differ among products, different fermented foods may inherently vary in the flexibility or constraints of their core microbiomes. Therefore, to achieve a more comprehensive understanding of functional dynamics within complex microbial communities, metagenomics must be combined with other molecular omics approaches, including metatranscriptomics, metaproteomics, and metabolomics.

### 2.2. Metatranscriptomics

Unlike metagenomics, which directly extracts and analyzes microbial DNA from samples to infer taxonomic composition and functional potential, metatranscriptomics captures the active state of microorganisms during fermentation by performing high-throughput sequencing and analysis of total RNA, especially messenger RNA (mRNA), in complex microbial communities. In this way, it reveals, at the transcriptional level, which microorganisms are functionally active and which biological processes are actually being expressed [1]. While metagenomics mainly reflects community composition and functional potential, metatranscriptomics focuses more on the real-time physiological state and active expression of microbial communities under specific environmental conditions. It therefore offers unique advantages for identifying functional executors, key metabolic pathways, and dynamic regulatory mechanisms in complex fermentation systems (Figure 2 and Figure 3) [58]. In recent years, multi-omics studies have generally regarded metatranscriptomics as a key layer linking community composition to metabolic phenotype, because it can more directly reflect microbial responses to raw-material characteristics, environmental stress, and changes across fermentation stages, thereby providing important evidence for elucidating the dynamic mechanisms of traditional fermentation. Despite these advantages, the application of metatranscriptomics in TFFB remains relatively limited, likely because RNA is highly susceptible to degradation under the extreme conditions often encountered in fermentation systems. In addition, the relatively high cost of metatranscriptomic analysis has also restricted its widespread adoption (Figure 2 and Figure 3) [1,53,59].

Fortunately, with the continuous improvement of sequencing technologies and the reduction in analytical costs, metatranscriptomics has gradually begun to find applications in TFFB, including Baijiu [60,61], vinegar [62], kimchi [63], and cheese [64]. In a recent study on Shanxi aged vinegar fermentation, researchers used metatranscriptomics to comprehensively investigate the active microbial community and key metabolic functions during the natural fermentation process. At the early stage of fermentation, *LAB*, *Acetobacter*, and *Pichia* were the dominant active microorganisms. As fermentation progressed, the active community gradually shifted toward one dominated by *L. helveticus*, *P. kudriavzevii*, *L. acetotolerans*, *A. pasteurianus*, and *A. oryzoeni* [62]. Further analysis showed that *A. pasteurianus*, *P. kudriavzevii*, *A. jinshanensis*, *A. oryzoeni*, *S. cerevisiae*, *L. helveticus*, *A. senegalensis*, and *L. acetotolerans* were identified as the core aroma-associated microbial species in Shanxi aged vinegar Cupei through metabolic network analysis and Pearson correlation analysis of key aroma-active compounds (KAACs), and that their succession was closely associated with KAAC production during vinegar fermentation [62].

Similarly, in a study investigating the microbial contributors to ester metabolism in medium-temperature Daqu, with esters being major flavor compounds in Baijiu, metatranscriptomic analysis revealed an active core microbiota predominantly composed of *Aspergillus*, *Pichia*, *Bacillus*, *Weissella*, and *Leuconostoc*, with *Aspergillus* playing a central functional role in ester formation. Subsequent fermentation validation further elucidated a multispecies ester biosynthetic network involving *A. flavus* T3, *Paenibacillus* sp. N1, and *Saccharomycopsis fibuligera* B3, with *Aspergillus* identified as the keystone genus driving ester production and flavor enhancement during Daqu fermentation [65]. These findings indicate that metatranscriptomics has considerable potential for supporting the reproducible production of fermented foods and beverages.

Although metatranscriptomics can reveal the functional state of complex microbial communities at the level of active gene expression, its application in TFFB still faces notable limitations [33,35,66,67]. First, RNA itself is inherently unstable and highly prone to degradation during sampling, storage, and extraction, especially in complex fermentation matrices characterized by high salt, high acidity, or abundant enzymes and polyphenols. This can directly compromise transcript integrity and the reliability of downstream sequencing results. Meanwhile, rRNA usually accounts for the vast majority of total RNA in samples, whereas mRNA represents only a small fraction. As a result, rRNA depletion is often required, but this step itself may introduce additional bias and affect transcript recovery efficiency across taxa. Second, the interpretation of metatranscriptomic data remains challenging (Figure 2). Compared with metagenomics, metatranscriptomics involves stronger temporal dynamics and relies more heavily on high-quality reference genomes and functional databases. For many bacteria, yeasts, and filamentous fungi in traditional fermentation systems that remain insufficiently studied, limited reference sequences, incomplete annotations, and a high proportion of hypothetical proteins can all restrict the accurate identification of functional genes and metabolic pathways. This problem is particularly pronounced for non-bacterial groups, such as fungi and archaea, for which database coverage and analytical tools remain less developed.

From a technical and practical perspective, metatranscriptomics also faces challenges including relatively high sequencing cost, substantial computational demands, and limited sensitivity for detecting low-abundance but active members [58,68,69]. Because transcript abundance can differ greatly among microorganisms in complex fermentation systems, highly expressed transcripts from dominant taxa may mask signals from rare but functionally important members, thereby hindering the identification of low-abundance active microorganisms.

In addition, current bioinformatics pipelines still face challenges in cross-species transcript quantification, homologous gene discrimination, and assignment of expression signals at the community level, which further limits the broader application of metatranscriptomics in industrial fermentation monitoring and routine quality control (Figure 2 and Figure 3) [58,66,70]. These limitations become more pronounced during production scale-up. Industrial fermentation systems usually involve larger volumes, stronger spatial heterogeneity, slower sampling feedback, and greater fluctuations in temperature, oxygen transfer, moisture, acidity, and substrate distribution. As a result, transcriptomic signals obtained from small-scale or laboratory fermentations may not fully represent the physiological state of microorganisms in large-scale tanks, solid-state piles, or open fermentation environments. In addition, industrial monitoring requires rapid, robust, and low-cost analytical workflows, whereas metatranscriptomics still depends on careful RNA preservation, high-quality extraction, sequencing, and complex data interpretation. These requirements make it difficult to use metatranscriptomics as a routine real-time control tool at production scale. Therefore, before broader industrial application, metatranscriptomic analysis should be integrated with representative sampling strategies, process sensors, fermentation kinetics, and metabolite monitoring to improve its scalability and practical value.

Moreover, although metatranscriptomics reflects functions that are being expressed, this does not necessarily correspond to final protein production or metabolite output. In other words, transcript levels do not always linearly correspond to actual metabolic activity, cellular physiological status, or terminal phenotypes, since enzymatic reactions ultimately occur at the protein level. Therefore, metatranscriptomic data alone are often insufficient to establish a complete causal chain linking active microorganisms, functional expression, flavor formation, and quality phenotype [1]. This limitation is particularly pronounced in fermented food systems, where multispecies cooperation and tightly coupled metabolism are common. Accordingly, metatranscriptomics generally needs to be integrated with metagenomics, metaproteomics, metabolomics, and culturomics in order to generate more reliable biological interpretations.

### 2.3. Metaproteomics

Research aimed at characterizing the overall protein profiles of microbial communities at specific time points has increased steadily since 2004, when the concepts of the metaproteome and metaproteomics were first proposed [1]. Metaproteomics refers to the large-scale separation, identification, and quantification of all proteins in complex microbial communities under specific environmental conditions and at defined time points, with the aim of revealing, at the protein-expression level, which functions are actually being executed within the community and which microorganisms are the direct contributors to ongoing metabolic activity (Figure 3) [71]. Unlike metagenomics, which mainly reflects community composition and functional potential, and metatranscriptomics, which reflects transcriptional activity, metaproteomics is more closely linked to the actual functional output of fermentation systems and is therefore regarded as a key layer connecting “genetic information” to “metabolic phenotype.” Recent reviews have highlighted that metaproteomics provides more direct functional information on complex microbial communities and offers unique advantages in identifying active functional microorganisms, elucidating substrate degradation processes, revealing flavor-formation mechanisms, and tracking microbial interactions in TFFB (Figure 2) [1,42]. Particularly under conditions of open fermentation, multispecies co-metabolism, and complex matrices, metaproteomics can identify key enzymes and functional proteins involved in carbohydrate utilization, protein hydrolysis, amino acid conversion, lipid degradation, organic acid formation, and volatile flavor compound biosynthesis, thereby more accurately revealing the actual contribution of microbial communities to product quality formation. Compared with function inference based solely on DNA or RNA, metaproteomics more directly reflects the biochemical reactions that actually occur during fermentation and thus has important value in clarifying functional division of labor and metabolic cooperation in traditional fermentation systems [70].

For example, during the spontaneous fermentation of maize dough, metaproteomics not only resolved changes in the composition of active microbial communities across different fermentation stages, but also linked dominant taxa such as *Actinobacteria*, *Proteobacteria*, and *Firmicutes* to key metabolic processes including carbohydrate degradation, glucose utilization, and starch conversion, thereby enabling a more direct analysis of the relationship among community succession, functional expression, and substrate transformation [72]. In studies of fermented vegetables, the combined use of metaproteomics and metagenomics has enabled the identification of key functional pathways related to carbohydrate and amino acid metabolism and linked these pathways to dynamic changes in dominant microbial taxa [73]. In Baijiu-related research, metaproteomics has been applied to analyze differences in the functional microbial community composition and carbohydrate-utilization capacity of Daqu produced across multiple production rounds. These analyses revealed that 23 fungal species and 5 bacterial species were involved in carbohydrate metabolism. Starch, cellulose, and glucan metabolism were identified as the main pathways, with fungi—especially *Aspergillus*—being more actively involved than bacteria. Among these active microorganisms, *Saccharomycopsis fibuligera*, *Aspergillus oryzae*, *Monascus purpureus*, *Byssochlamys spectabilis*, *Lichtheimia ramosa*, *Thermomyces lanuginosus*, and *Thermoascus aurantiacus* were identified as important functional microorganisms with the capacity to produce multiple enzymes [74]. In addition, saccharifying power is usually regarded as an indicator of Daqu quality; however, based on metaproteomic analysis and fermentation validation, saccharifying activity was found not to be consistently positively correlated with alcohol yield in Baijiu fermentation [75].

Overall, metaproteomics overcomes the limitations that “presence does not equal activity” and “transcription does not equal functional realization,” thereby enabling the identification of key active microorganisms and their metabolic tasks in traditional fermentation systems from the perspective of functional execution. Accordingly, this technique has gradually become an important tool for studying the functional mechanisms of microbial ecology in TFFB and is often used in combination with metagenomics, metatranscriptomics, and metabolomics to establish a more complete framework linking community composition, functional expression, metabolic products, and quality phenotype [39].

Nevertheless, the application of metaproteomics in TFFB still faces substantial limitations. First, fermented food matrices are typically complex and rich in starch, proteins, polysaccharides, lipids, salts, polyphenols, and various small-molecule metabolites, all of which can significantly interfere with protein extraction, purification, and subsequent mass spectrometric detection [76,77]. Protein extraction remains one of the major challenges in food fermentation metaproteomics, and standardized extraction protocols specifically tailored to fermented food systems are still limited [71,78,79]. Second, metaproteomic data analysis is highly dependent on the quality of reference databases. Traditional fermentation systems often contain many bacteria, yeasts, and filamentous fungi that remain insufficiently studied, and the lack of high-quality reference genomes or protein databases can result in low protein-identification rates, difficulty in distinguishing homologous proteins, and inaccurate functional annotation [42]. Because protein abundances can vary greatly among microorganisms in complex fermentation communities, proteins derived from dominant taxa may mask those from low-abundance but functionally important members, thereby hindering the identification of core functional microorganisms. Recent reviews have highlighted database dependence, sample complexity, and the difficulty of detecting low-abundance proteins as major constraints in the application of metaproteomics to food fermentation research [71]. In general, metaproteomics is a powerful tool for compensating for the limitations of metagenomics and metatranscriptomics at the level of functional execution and for deciphering the actual metabolic activities of microbial communities in TFFB; however, bottlenecks remain in sample pretreatment, protein-identification coverage, and annotation accuracy.

Moreover, although metaproteomics is closer to actual functional output than metagenomics or metatranscriptomics, it still cannot be considered fully equivalent to ultimate metabolic fluxes or final product phenotypes. The detection of a protein does not necessarily mean that its catalytic efficiency, post-translational modification status, or actual metabolic contribution has been fully clarified. Therefore, in research on TFFB, metaproteomics still needs to be integrated with metagenomics, metatranscriptomics, and metabolomics in order to more completely establish the relationship among community composition, functional expression, metabolic products, and quality attributes.

### 2.4. Metabolomics

Metabolomics is an analytical approach for detecting metabolites in a target system. The small-molecule metabolites analyzed include a wide range of endogenous and exogenous compounds, such as peptides, amino acids, nucleic acids, carbohydrates, organic acids, vitamins, polyphenols, alkaloids, minerals, and any other substances that cells or organisms can utilize, ingest, or synthesize (MW ≤ 1700 Da) [80]. Larger molecules, such as DNA, RNA, and proteins, are excluded from metabolomics datasets. Compared with metagenomics, which mainly reveals what is present in the community and what it may be capable of doing, and with metatranscriptomics and metaproteomics, which reflect which genes are being expressed and which functions are being executed, respectively, metabolomics is more directly linked to the final metabolic phenotype of the fermentation process and is therefore regarded as a key layer connecting microbial functional activity with the sensory, nutritional, and functional attributes of the final product (Figure 2 and Figure 3) [81,82].

Methodologically, metabolomics generally includes sample pretreatment, metabolite extraction, separation and detection, data preprocessing, statistical analysis, and metabolic pathway interpretation [83]. Depending on the research objective, metabolomics can be divided into untargeted and targeted metabolomics. The former is suitable for comprehensively profiling the metabolome and identifying differential biomarkers and potential key metabolites, whereas the latter is more appropriate for the highly sensitive and accurate quantification of specific metabolites [83]. At present, nuclear magnetic resonance (NMR) and mass spectrometry-based platforms are the most widely used techniques in fermented food and beverage research. Among them, NMR offers advantages in simple sample preparation, good reproducibility, and strong structural elucidation capability, whereas GC-MS, LC-MS, and UHPLC-QTOF-MS provide superior performance in terms of metabolite coverage, sensitivity, and the qualitative and quantitative analysis of flavor compounds [83,84,85,86,87]. In addition to these platforms, Fourier-transform infrared spectroscopy (FTIR) has also been widely used as a rapid, non-destructive, and high-throughput approach for metabolic fingerprinting, especially when combined with chemometric or multivariate statistical analysis [88]. Although FTIR generally provides lower structural specificity and metabolite annotation depth than NMR- and MS-based platforms, it can capture global spectral changes associated with major biochemical groups and is therefore useful for process monitoring, sample discrimination, and quality evaluation in fermented foods and beverages [88,89].

In research on TFFB, metabolomics can directly characterize the generation, accumulation, transformation, and consumption of metabolites during fermentation, thereby providing direct evidence for elucidating flavor formation, quality differentiation, and the enrichment of functional compounds. Recent reviews have indicated that metabolomics has been widely applied to fermented soybean products, dairy products, alcoholic beverages, fermented vegetables, fish sauce, vinegar, and tea products, and can be used to identify key flavor compounds, compare metabolic differences under different microbial strains or processing conditions, and mine bioactive compounds associated with antioxidant, antihypertensive, and immunomodulatory properties [81,90,91,92].

Metabolites in TFFB, such as sugars, amino acids, organic acids, and volatile compounds, can profoundly influence taste and flavor. Most flavor compounds are secondary metabolites derived from food components and are typically characterized by low molecular weight, low abundance, and high volatility. Metabolomics can comprehensively analyze the composition and concentration of these volatile substances, thereby helping optimize food production and processing to improve the flavor quality of final products. Even within the same Daqu-making room, spatial and temporal differences in physicochemical factors, such as temperature, can give rise to black, yellow, and white Daqu during high-temperature Daqu production. Based on untargeted metabolomics, five-membered heterocyclic amino acids were identified as the major factors underlying the differences among these three Daqu types [93]. Further validation experiments showed that *Neurospora crassa*, *Aspergillus nidulans*, *Bacillus subtilis*, and *Oceanobacillus iheyensis* were the key microorganisms driving microecological differentiation in high-temperature Daqu [94].

During fermented tea production, leaf tenderness markedly influences the taste and aroma of the final product. By combining sensory evaluation, electronic tongue analysis, and metabolomics, researchers showed that black tea prepared from more tender leaves exhibited stronger sweetness, umami, and fruity aroma, together with reduced bitterness. Metabolomic analysis identified 519 differential taste-related compounds and 84 differential aroma compounds. Increased levels of maltitol, cycloleucine, L-methionine, and L-aspartic acid were associated with enhanced sweetness and umami, whereas decreased levels of 4-aminosalicylic acid, tyrosol, uracil, inosine, benzamide, and L-tyrosine contributed to reduced bitterness. In addition, elevated levels of volatile compounds such as (Z)-2-decenal, propyl acetate, acetophenone, and 2-phenylethanol promoted fruity aroma formation [95]. These findings provide a theoretical basis for grading black tea flavor quality according to leaf tenderness.

Terroir is a major factor shaping grape microecology and the final flavor composition and quality of wine [55,96,97]. Metabolomics can reveal region-dependent differences at the chemical phenotype level and provide molecular evidence for terroir characteristics. In Cabernet Sauvignon grapes and wines from five grape-producing regions in China, UHPLC-QqQ-MS/MS combined with multivariate and pathway analyses identified 94 anthocyanins and their derivatives, 78 non-anthocyanin phenolics, and a series of key differential metabolites, including flavonols, stilbenes, hydroxycinnamic acids, and peonidin- and malvidin-derived compounds. Some anthocyanins and flavonols were significantly correlated with climatic factors such as temperature, precipitation, and relative humidity; for example, malvidin-3-O-glucoside was positively associated with temperature, whereas several metabolites were negatively associated with precipitation [98]. These differences were mainly enriched in flavonoid biosynthesis, indicating that terroir may shape wine color, phenolic composition, and sensory style by modulating related metabolic branches. Thus, metabolomics is valuable not only for origin discrimination and biomarker screening, but also for elucidating the mechanisms through which terroir drives wine quality formation [99].

Despite its clear advantages in elucidating flavor formation, quality differences, and the accumulation of functional compounds in TFFB, metabolomics also has notable limitations. The chemical complexity of fermented matrices and the diverse physicochemical properties of metabolites make comprehensive coverage difficult to achieve with a single extraction method or analytical platform. In untargeted analyses, a high proportion of unknown metabolites, together with incomplete databases and limited standards, constrains the accuracy of metabolite annotation and quantification (Figure 2). As a result, metabolomics can often reveal what is produced, but is less able to explain why it is produced, by whom, and how it is regulated. Moreover, metabolomic profiles are highly sensitive to raw materials, processing conditions, sampling time, and storage status, which limits comparability and reproducibility across studies [81,83,90,100]. Finally, although metabolomics is closely linked to terminal phenotypes, changes in metabolites alone cannot directly establish causal relationships with microbial composition, gene expression, or protein function [87]. Moreover, a persistent challenge in current metabolomics is that relationships determined using correlation-based methods, such as Pearson or Spearman correlations, may fail to accurately capture biological meaning and often do not adequately account for the complexity of microbial interactions [101]. Most studies also lack experimental validation and fail to reproduce observed or inferred relationships using culture-based approaches. A more comprehensive strategy for validating such relationships should include, for example, reproducing specific food characteristics in fermentations inoculated with particular isolated strains relative to appropriate controls.

### 2.5. Culturomics

Culturomics is a high-throughput cultivation strategy that has emerged in recent years. By combining diversified media, differentiated cultivation conditions, rapid strain identification, and large-scale isolation and screening, it seeks to recover as many microbial strains as possible from complex microbial ecosystems, thereby compensating for the limitations of traditional culture-based methods, which typically yield a low cultivability rate, and high-throughput sequencing, which can often detect microorganisms but cannot readily validate them experimentally (Figure 2) [102]. Culturomics is not a single technique, but rather a systematic framework integrating expanded cultivation conditions, automated isolation, identification by MALDI-TOF MS or sequencing, strain preservation, and functional validation. It was first systematically proposed in human microbiome research and has since gradually expanded to soil, food, and other complex microbial ecosystems [102,103,104].

Unlike metagenomics, metatranscriptomics, metaproteomics, and metabolomics, which focus, respectively, on community composition, transcriptional activity, functional execution, and terminal metabolic phenotype, the unique advantage of culturomics lies in its ability to directly obtain living and manipulable microbial resources [105]. Therefore, culturomics is regarded as an important bridge linking “omics discovery” with “experimental validation” and as a key means of advancing microbiome research from descriptive analysis toward mechanistic interpretation and engineering application (Figure 3).

In TFFB research, many potential metabolic traits of fermentation-associated microorganisms remain unverified by culture-based approaches, making culturomics particularly valuable for elucidating the functions of previously uncultured taxa. Because these systems are often characterized by open fermentation, multispecies coexistence, complex microbial interactions, and strong environmental stress, sequencing alone is often insufficient to identify truly usable functional microorganisms or to verify their specific roles in flavor formation, substrate degradation, acid and alcohol production, enzyme secretion, or safety control. By expanding cultivation conditions, culturomics enables more effective isolation of low-abundance, slow-growing, and nutritionally fastidious microorganisms, thereby establishing active strain collections that support functional strain screening, starter development, and synthetic consortium construction [106,107]. Recent reviews further suggest that combining culturomics with metagenomics can more comprehensively reveal both the “detectable” and “usable” diversity of complex microbial ecosystems, thus providing important methodological support for functional strain mining, key microorganism validation, and the development of reproducible fermentation systems in TFFB [53,102,108].

In the field of TFFB, recent studies have accumulated substantial evidence supporting the application potential of culturomics. For example, by using more than 20 different cultivation conditions, researchers isolated 19 novel strains from Baijiu-related systems that had not been reported in previous Baijiu studies [109]. Similarly, by applying two cultivation temperatures and four media, more than 400 strains were successfully isolated from naturally fermented milk, including four strains that had not been previously reported [110]. In a more systematic study, 56 enrichment conditions generated from multiple media, redox states, incubation times, and selective inhibitors enabled the recovery of more than 90 strains, including members of *Weissella*, *Bacillus*, and *Lactococcus*, and substantially expanded the traditional *LAB*-centered strain library [111]. Beyond TFFB, culturomics has also been widely applied in rhizosphere microbiology [102] and gut microbiology [103,112]. Taken together, these findings indicate that culturomics can substantially expand strain repositories and thereby increase the microbial resources available for constructing synthetic microbial consortia in food fermentation.

Although culturomics markedly improves the recoverability of microorganisms from complex fermentation systems and provides living strains for downstream functional validation, it still has important limitations. As a cultivation-dependent strategy, it is inherently subject to selective bias imposed by cultivation conditions and therefore cannot fully reconstruct the original microbial structure of a sample. Rare taxa, slow-growing microorganisms, oligotrophs, VBNC microorganisms, and members dependent on community interactions are often difficult to recover effectively using conventional cultivation systems [31]. In addition, culturomics is time-consuming, labor-intensive, and less suitable for large-scale studies, while colony selection and post-cultivation identification may also introduce bias or uncertainty. More importantly, it captures only the cultivable and isolable fraction of the community rather than all active members, and therefore cannot independently resolve the true ecological roles and functional contributions of the entire microbiota. For this reason, culturomics is better regarded as a complement to high-throughput sequencing and other multi-omics approaches rather than a standalone source of evidence. Moreover, effective cultivation strategies should be guided by a broader understanding of microbial metabolic traits, which in turn requires extensive mining of metagenomic, metatranscriptomic, metaproteomic, and metabolomic datasets.

### 2.6. Why and How Can Multi-Omic Approaches Be Combined?

Traditional fermented foods are essentially complex dynamic systems jointly shaped by community composition, functional expression, metabolic products, and quality phenotypes. A single omics approach can usually capture only one layer of this system. For example, metagenomics mainly reveals which microorganisms are present and what their potential functions may be; metatranscriptomics identifies which genes are being expressed and which microorganisms are metabolically active; metaproteomics elucidates which proteins are actually executing functions and which microorganisms are responsible for them; and metabolomics more directly reflects the final metabolites that are produced [113,114].

However, relying on a single omics approach is insufficient to fully explain the intrinsic links among microbial succession, substance transformation, and flavor and quality formation. This limitation is even more pronounced in research on synthetic microbial consortia, because consortium construction requires not only the identification of core functional microorganisms, but also clarification of their interactions, metabolic division of labor, and contributions to final product characteristics. Therefore, when designing and constructing reproducible and controllable synthetic microbial consortia for traditional fermented products with target properties, it is necessary to integrate high-throughput sequencing, including both DNA- and RNA-based approaches, with metaproteomics and metabolomics in order to comprehensively characterize microbial composition and dynamics, enzyme functions, and metabolic phenotypes throughout fermentation [33,35].

More specifically, the value of combining multi-omics approaches lies in the ability to connect information from different biological layers into a continuous chain of evidence. In general, metagenomics is used to characterize community composition and functional potential; metatranscriptomics reveals actively expressed genes and pathways; metaproteomics confirms key functional proteins and their actual execution processes; and metabolomics depicts terminal metabolites and their relationships with flavor, nutrition, and safety. In addition, culturomics can further convert omics findings into manipulable living microbial resources that can be used for functional validation and community reconstruction. Through such integration, a more systematic framework can be established to link community composition, functional expression, metabolic products, and quality phenotype. In earlier studies, metagenomics or amplicon sequencing was commonly used to reveal microbial community composition in fermentation systems, thereby yielding microbial relative abundance data. Core microbiota were then often selected using either an abundance threshold greater than 1% or network module analysis [9]. However, high abundance does not necessarily mean that a strain makes a proportionally greater contribution to the fermentation system, because some low-abundance taxa may exert disproportionately large effects on community composition, function, and stability [9,115]. To address this limitation, multiple omics approaches are often employed for cross-validation.

For instance, in a study on naturally fermented sourdough, combined metagenomic and metatranscriptomic analyses showed that the community was not determined solely by highly abundant dominant taxa, but instead displayed a hierarchical structure composed of dominant, subdominant, and satellite species, with different tiers participating in distinct metabolic pathways [116]. More importantly, based on these findings, the researchers further reconstructed a synthetic sourdough consortium and demonstrated that retaining only the dominant species was insufficient to maintain system resilience and performance, whereas subdominant and satellite species also made irreplaceable functional contributions [116]. Similarly, in studies of Chinese Daqu, the combined analysis of metagenomics, metatranscriptomics, and metaproteomics showed that although metagenomics detected 5211 microbial species, only 1774 were actually active, indicating a clear distinction between being “detected” and truly “functioning.” By integrating functional analysis with isolation-based validation, the researchers further identified specific functional microorganisms associated with the formation of lactic acid, pyrazines, and phenylethanol [117].

In vinegar fermentation research, the combination of microbiomics and metabolomics has further demonstrated the complementary advantages of multi-omics. On the one hand, metagenomics or high-throughput sequencing can be used to analyze community succession and functional potential; on the other hand, GC-MS, LC-MS, and other metabolomics tools can be used to identify volatile and non-volatile flavor compounds. Correlation analysis can then be applied to pinpoint core functional microorganisms closely associated with the formation of key metabolites, thereby directly linking community change to flavor output [118].

Beyond conventional omics data, some researchers have also adopted deep learning-based methods to screen core microorganisms in environmental systems [119,120], and recent studies on Daqu microbiota have successfully validated the reliability of this approach [121]. These findings suggest that the integration of multi-omics data with advanced computational approaches may further improve the identification of functionally important microorganisms and strengthen the rational design of synthetic microbial consortia.

Taken together, multi-omics integration provides a stepwise route from microbial detection to functional validation and DMC reconstruction. Metagenomics first defines the detectable community and its functional potential, while metatranscriptomics narrows this pool to microorganisms and pathways that are active during fermentation. Metaproteomics further verifies whether these functions are translated into enzymes and metabolic tasks, and metabolomics links microbial activity to flavor, nutritional, safety, and quality phenotypes [102,107,122,123,124]. Culturomics then converts candidate microorganisms identified by omics analyses into living strains that can be experimentally tested. Therefore, the value of multi-omics is not simply the accumulation of independent datasets, but the construction of a continuous evidence chain linking “who is present”, “who is active”, “what functions are executed”, “what metabolites are produced”, and “which strains can be reconstructed into a controllable consortium” [42]. This integrated logic provides a practical basis for selecting keystone and helper microorganisms, validating microbial interactions, and iteratively optimizing DMCs through the Assembly-Assessment-Redesign framework (Table 1).

## 3. The Bipartite Structure of DMCs

**Figure 4:**
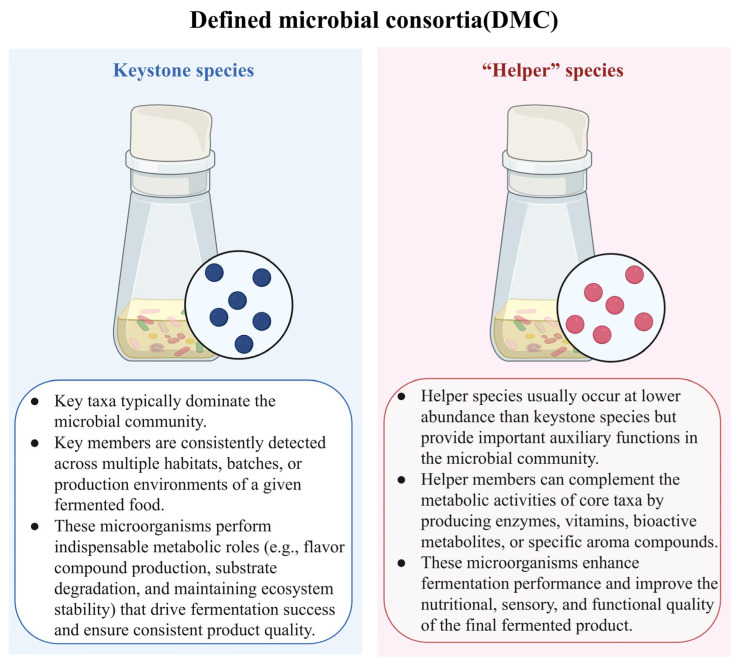
The bipartite structure and definition of DMCs.

Although multi-omics technologies have greatly improved our ability to resolve microbial composition, functional potential, active expression, and metabolic output in traditional fermentation systems, such information must still be translated into an operational framework before it can effectively guide rational DMC construction. Recent studies in fermented food microbial ecology increasingly suggest that the stability, functionality, and typicity of complex fermentation systems do not arise from the average contribution of all community members, but rather from the coordinated action of two functionally distinct groups of microorganisms. One group plays dominant roles in community assembly, key substrate conversion, and core metabolic output, whereas the other facilitates the colonization, growth, and functional expression of the former through nutritional cross-feeding, environmental modulation, stress buffering, and metabolic complementation. The former can be described as the keystone microbiome, whereas the latter can be further defined as the helper microbiome (Figure 4) [32,125]. In this context, the keystone microbiome determines the overall direction of fermentation, whereas the helper microbiome determines whether that direction can be achieved in a stable, efficient, and reproducible manner. Accordingly, from the perspective of DMC design, conceptualizing traditional fermentation systems as a bipartite structure composed of a keystone microbiome and a helper microbiome is not only more consistent with current ecological understanding of key taxa and functional interactions, but also more conducive to translating multi-omics information into practical strategies for microbial screening, community assembly, and iterative optimization [6,115].

### 3.1. The “Keystone Microbiome”

A large body of evidence has demonstrated that core microorganisms in traditional fermented food and beverage systems play crucial roles in community functioning [51,126,127,128,129]. In this context, we propose a related but more function-oriented concept, namely the keystone microbiome, and define keystone species as one or several microbial strains that are central to community assembly and/or to the execution of specific functions, such as substrate degradation, flavor formation, and the suppression of spoilage microorganisms. For example, *LAB*, *Pichia*, and *Rhizopus* are important contributors of saccharifying enzymes and glycosyltransferases in Jiuqu [130], and these enzymes facilitate the conversion of starch-rich substrates.

From the perspective of dominant metabolic routes and ecological selection pressures, DMCs in traditional fermented foods can be broadly classified into four major types: lactic, acetic, alcoholic, and alkaline fermentation systems [131,132,133]. Although these four systems differ substantially in substrate conversion pathways, end-product characteristics, and environmental trajectories, they share one common feature: they are not driven by a single high-abundance species, but are instead maintained by a set of microbial members that play pivotal roles in metabolic driving forces, ecological filtering, and quality formation.

Lactic fermentation systems are characterized by the dominance of lactic acid bacteria, which rapidly produce lactic acid through carbohydrate metabolism, lower the pH, suppress spoilage and pathogenic microorganisms, and simultaneously promote flavor, texture, and product preservation. In such systems, the keystone microbiome generally includes *Leuconostoc*, *Weissella*, *LAB*/*Lactiplantibacillus*, and *Pediococcus*, although different members may play different roles at different stages. Early colonizers are more closely associated with initial establishment and flavor foundation, whereas acid-tolerant taxa tend to dominate sustained acidification and community stabilization at later stages [133,134]. Taking kimchi as an example, both original studies and reviews have shown that its fermentation is primarily driven by lactic acid bacteria, among which *Leuconostoc*, *Weissella*, and *LAB* are the most common keystone groups; in particular, *Leuconostoc* is often associated with early dominance, flavor improvement, and quality enhancement [134]. Another representative example is sourdough, whose keystone microbiome typically exhibits a lactic acid bacterium–yeast cooperative structure, in which *Fructilactobacillus sanfranciscensis*, *Kazachstania humilis*, and *Saccharomyces cerevisiae* collectively contribute to acidification, gas production, and aroma formation, reflecting the clear division of labor characteristic of lactic fermentations [135].

Acetic fermentation systems are defined by the oxidation of ethanol to acetic acid, and their keystone microbiome is generally centered on acetic acid bacteria, mainly including *Acetobacter*, *Komagataeibacter*, and *Gluconobacter*. These microorganisms not only determine acid accumulation efficiency and fermentation intensity, but also directly influence the acidic aroma profile and stress tolerance of the system. Importantly, acetic fermentation is not carried out by acetic acid bacteria alone, but rather by a cooperative network involving yeasts and lactic acid bacteria: yeasts provide ethanol and volatile precursors, whereas lactic acid bacteria contribute to environmental modulation, substrate turnover, and community stabilization [136]. The most representative example is vinegar fermentation. In traditional vinegar systems, acetic acid bacteria are the direct executors of ethanol oxidation, while yeasts and lactic acid bacteria jointly shape fermentation ecology and flavor complexity. Recent studies and reviews consistently emphasize that vinegar fermentation should be regarded as an AAB–yeast–LAB co-driven ecosystem rather than a single-species acetic acid fermentation [137,138]. Another representative example is kombucha. This system is typically driven by a symbiotic culture of bacteria and yeast (SCOBY), in which yeasts first convert sugars into ethanol, and *Komagataeibacter* and related acetic acid bacteria subsequently oxidize ethanol to acetic acid, a process closely associated with the accumulation of acidic metabolites such as gluconic acid. Studies have further shown that increases in *Komagataeibacter* abundance are significantly associated with decreases in pH during kombucha fermentation [139,140,141,142,143].

Alcoholic fermentation systems are primarily oriented toward ethanol production, and their keystone microbiome is typically built around ethanol-producing yeasts, with *Saccharomyces cerevisiae* being the most common central driver because of its strong sugar metabolism, ethanol tolerance, and ecological competitiveness. However, in many traditional alcoholic fermented foods and beverages, the true keystone microbiome is not restricted to *Saccharomyces*, but instead consists of a hierarchical functional network formed by *Saccharomyces* together with non-*Saccharomyces* yeasts, lactic acid bacteria, and even saccharifying filamentous fungi [133]. In wine fermentation, for example, although *S. cerevisiae* usually dominates the main fermentation, early stages often involve non-*Saccharomyces* yeasts such as *Candida*, *Hanseniaspora*, *Issatchenkia*, *Kluyveromyces*, *Metschnikowia*, and *Pichia*, which make important contributions to aroma precursor release and sensory complexity. Recent studies have further shown that the combined use of *Torulaspora delbrueckii*, *Hanseniaspora uvarum*, and *S. cerevisiae* can markedly alter the chemical and sensory properties of Sauvignon blanc [144,145]. Another representative example is Baijiu fermentation. In sauce-flavor Baijiu, multiple non-*Saccharomyces* yeasts, in addition to *S. cerevisiae*, have been shown to participate in higher alcohol formation and flavor regulation, indicating that the keystone microbiome in alcoholic fermentation systems often consists of both a “core ethanol-producing module” and an “auxiliary aroma-forming module” [146].

Alkaline fermentation differs fundamentally from the above three types in that the process is usually accompanied by intensive protein hydrolysis and ammonia release, causing the system pH to rise rather than decline [147]. Such fermentations are widely found in traditional Asian and African foods based on legumes, seeds, or leaves, and their keystone microbiome is typically centered on *Bacillus*, especially *Bacillus subtilis* and its close relatives. Unlike lactic fermentation, which mainly relies on acidification for preservation, alkaline fermentation emphasizes protein degradation, the release of amino acids and peptides, the development of strong characteristic flavors, and enhanced nutrient accessibility [148,149]. Typical examples include Japanese natto, South Asian kinema, and West African dawadawa/iru. These products are usually based on protein-rich plant substrates, and their fermentation is often dominated by *B. subtilis*, with characteristic increases in pH, intensified ammonia odor, and extensive protein hydrolysis. Reviews have also noted that bacilli in alkaline fermented foods may not only contribute to texture and flavor, but may also enhance viscosity and confer certain health-promoting properties through metabolites such as poly-γ-glutamic acid [148,149].

Our understanding of the functional roles of these keystone microbiome members has, to a large extent, been derived from systematic investigations using multi-omics approaches. Overall, lactic, acetic, alcoholic, and alkaline fermentation systems correspond to four major metabolic axes: acid production, ethanol oxidation, ethanol generation, and alkaline protein degradation, respectively. The ecology and quality formation of traditional fermented foods are essentially driven by organic acids, ethanol, and other metabolic end products, and the major microbial groups involved in these processes usually include lactic acid bacteria, acetic acid bacteria, bacilli, yeasts, and filamentous fungi. In particular, in alkaline fermentation systems, Bacillus-dominated protein hydrolysis and ammonia release elevate pH and generate metabolic characteristics distinct from those of acidic and alcoholic fermentations. Therefore, in DMC design, understanding these four fermentation types should not stop at identifying the most abundant taxa, but should instead focus on identifying which members truly drive metabolic flow, maintain community stability, and shape final product quality. In this sense, the term keystone microbiome better captures their functional essence than the more abundance-oriented concept of a core microbiome [9,125,136,148].

Ultimately, within each functional group, only a limited number of functionally dominant microorganisms continuously participate in and support the core processes of a given fermented food. Based on this understanding, multi-omics approaches can jointly resolve the core microbiomes of different fermented foods at the species or even strain level, thereby simultaneously capturing community composition, functional potential, active expression, and metabolic output. Such integrated analysis is crucial for identifying the key microorganisms and dominant metabolic pathways that sustain fermentation and can therefore provide direct guidance for the rational construction of DMCs. In wine, for example, multi-omics studies have shown that non-*Saccharomyces* yeasts such as *Starmerella* and *Hanseniaspora* not only participate in community succession during spontaneous fermentation, but are also closely associated with metabolic characteristics; further integration of synthetic must fermentation, transcriptomics, and metabolic profiling has shown that dominant yeast species can determine fermentation performance and final metabolite composition, thus providing a basis for the design of targeted mixed fermentations [150,151]. In contrast, Baijiu research has already developed a more complete “identification–validation–reconstruction” pathway. For example, in Xiaoqu light-aroma Baijiu, researchers first identified 10 core microorganisms and then validated through simulated fermentation that these core members could reproduce the traditional fermentation process and product flavor [47]; Likewise, in sauce-flavor Baijiu and Baobaoqu studies, multi-omics further revealed the distinction between “detected members” and “truly active members,” and identified specific functional microorganisms associated with key flavor formation [47,117,152]. These findings clearly indicate that the real value of multi-omics lies in advancing microbiome identification from simple abundance-based judgment to function-oriented interpretation and experimental validation, thereby directly supporting the precise design and reconstruction of DMCs.

### 3.2. The “Helper Microbiome”

Unlike the keystone microbiome, which directly drives substrate conversion and end-product formation, the helper microbiome refers to those auxiliary members that may not themselves be the primary executors of metabolism and may not always occur at high abundance, yet can nonetheless promote the colonization, growth, and functional expression of key microorganisms through nutritional cross-feeding, environmental regulation, stress buffering, substrate pretreatment, or metabolic complementation [125,153]. From an ecological perspective, the core role of these microorganisms does not lie in directly determining the yield of target products, but rather in improving the habitat conditions of key taxa, thereby enhancing community establishment efficiency, process stability, and system resilience. Therefore, the helper microbiome can be regarded as an “ecological facilitating layer” that supports the function of the keystone microbiome. Its functions typically include providing utilizable substrates or growth factors, removing inhibitory metabolites, regulating pH and redox status, improving attachment or biofilm formation, and enhancing the adaptability of key microorganisms to environmental fluctuations [125,154,155,156].

In TFFB, the importance of the helper microbiome is often underestimated, because these members may not dominate in abundance, yet they can substantially influence whether key microorganisms are able to successfully colonize and express their functions. In other words, if the keystone microbiome determines the overall direction of fermentation, then the helper microbiome often determines whether that direction can be stably realized [9,157]. Therefore, in DMC design, in addition to screening key functional microorganisms, it is also necessary to identify those auxiliary members that can enhance the survival, metabolic efficiency, and ecological stability of the key taxa.

In kimchi fermentation, *Leuconostoc mesenteroides* is often regarded as an important early-stage member. Its role lies not only in acid production itself, but also in its ability to rapidly establish an acidic and relatively anaerobic environment at the early stage of fermentation, thereby creating suitable conditions for the subsequent colonization of more acid-tolerant taxa such as *Levilactobacillus brevis* and *Lactiplantibacillus plantarum*. In this sense, *L. mesenteroides* displays a clear helper-like role during community succession, facilitating the successful establishment and replacement of subsequent key lactic acid bacteria and ultimately contributing to the maintenance of fermentation progression and product quality [158].

In kombucha and other acetic fermentation systems, yeasts can typically be regarded as representative members of the helper microbiome. Relevant studies have shown that yeasts hydrolyze sucrose by secreting invertase, thereby releasing glucose and fructose and subsequently producing ethanol; acetic acid bacteria then utilize these monosaccharides and ethanol to generate acetic acid and drive subsequent fermentation [159]. Therefore, in the absence of yeasts with strong sucrose-hydrolyzing and fermentative capacities, acetic acid bacteria may fail to obtain sufficient substrates, and both system acidification and biofilm formation may be impaired. This indicates that, in such fermentations, yeasts may not be the dominant drivers of final acid production, but they significantly promote the growth and functional expression of acetic acid bacteria through substrate supply and environmental shaping, and thus represent typical helper members [159].

In wine fermentation, the promoting role of non-*Saccharomyces* yeasts toward key microorganisms is mainly reflected in two aspects. First, they can alter the metabolic environment of the fermentation matrix, thereby affecting the performance of subsequent *Saccharomyces cerevisiae* or *Oenococcus oeni*. Second, they can reduce certain inhibitory factors and thus create more favorable conditions for downstream fermentation. Previous studies have shown that, in some sequential inoculation systems, *Torulaspora delbrueckii* or *Metschnikowia pulcherrima* can reduce SO_2_ and medium-chain fatty acid levels in wine, thereby facilitating malolactic fermentation (MLF) conducted by *Oenococcus oeni*; in some cases, faster MLF progression has even been observed [139,160]. These findings suggest that certain non-*Saccharomyces* yeasts are not merely co-fermenting members, but may also function as helper microbiome members that assist key lactic acid bacteria in establishing themselves and performing subsequent functions.

In Baijiu fermentation, the role of the helper microbiome is more strongly reflected in its support of key aroma-producing members and the core fermentation network. Recent reviews have pointed out that Baijiu fermentation is not completed by a few core microorganisms alone, but is jointly driven by complex interactions among molds, yeasts, and bacteria, in which many non-dominant members influence the functional output of the core microbiota through precursor release, metabolic complementation, and environmental regulation. Further empirical studies have shown that, in Chi-flavor Baijiu fermentation, the exogenous addition of *Pichia anomala* and *Lpb. paraplantarum* significantly increased the levels of alcohols and esters in the system, as well as key flavor compounds such as phenylethanol and ethyl lactate, indicating that these microorganisms can act as helper strains to strengthen the core aroma-producing network and improve fermentation performance [153,161]. Although they may not always be the most central executors of metabolism, they can nonetheless enhance the overall performance of key microbial groups through community reshaping and metabolic facilitation, and therefore exhibit clear helper attributes.

For DMC design, the keystone, and helper microbiomes should be distinguished using operational rather than purely descriptive criteria. The core microbiome refers to microorganisms that are consistently detected across fermentation batches, stages, regions, or production conditions and are therefore associated with the basic identity of a fermentation system. However, core members are not necessarily keystone members. The keystone microbiome should be further defined by measurable functional evidence, including high functional activity in metatranscriptomic or metaproteomic datasets, strong contribution to target metabolites or quality phenotypes, central roles in interaction or metabolic networks, and marked effects on community performance after perturbation, removal, or reintroduction. By contrast, the helper microbiome refers to members that may not independently determine the target phenotype, but can enhance the growth, activity, stress tolerance, colonization, or metabolic performance of keystone microorganisms through nutritional cross-feeding, environmental buffering, metabolic complementation, or community stabilization.

Overall, the core value of the helper microbiome lies in amplifying the function of key microorganisms rather than replacing them. Through nutritional cross-feeding, environmental buffering, metabolic complementation, and the maintenance of community stability, these members provide the keystone microbiome with a more favorable ecological niche and a more efficient functional background. Therefore, the assignment of a microorganism to the core, keystone, or helper category should be based on the level of supporting evidence. Co-occurrence, correlation with metabolites, or repeated detection across samples can be used to propose candidate core or candidate functional members, but these data alone should not be overinterpreted as causal evidence. Stronger evidence should come from co-culture assays, synthetic community reconstruction, strain-removal or strain-addition experiments, perturbation tests, and fermentation validation showing that the member changes community stability, target metabolite production, or sensory and quality outcomes. For DMC design, this means that the objective should not be limited to screening a few high-abundance or highly functional strains, but should also include identifying those auxiliary members that promote the colonization, growth, and coordinated expression of key taxa. Only when both association-based omics evidence and experimental validation are considered together can the keystone/helper framework be transformed from a conceptual model into a practical strategy for DMC assembly and optimization. Only by retaining both the keystone microbiome and the helper microbiome can reconstructed communities more closely approximate traditional fermentation systems in terms of stability, flavor complexity, and reproducibility [153].

## 4. Multi-Omics-Guided Design and Refinement of the DMCs

Although multi-omics technologies have substantially improved our ability to decipher microbial composition, functional potential, active expression, and metabolic output in traditional fermentation systems, such information must still be translated into practical design strategies before it can effectively support the construction and optimization of DMCs. In essence, the development of multi-omics-guided DMCs involves at least two closely connected steps. The first is the identification, on the basis of cross-level evidence, of the keystone microbiome, which plays dominant roles in community assembly, key metabolic processes, and quality formation, as well as the helper microbiome, which supports the stable expression of these functions through nutritional cross-feeding, environmental regulation, and functional complementation. The second is the subsequent optimization of microbial composition and interaction patterns through rational assembly, systematic assessment, and iterative redesign, with the ultimate aim of obtaining reconstructed fermentation systems that are stable, controllable, and functionally target-oriented. Accordingly, this section first discusses the logic for identifying keystone and helper microbiomes and then introduces the Assembly-Assessment-Redesign (A-A-R) framework for DMC optimization.

### 4.1. The Identification of Key and Helper Microbiomes

In the multi-omics-guided design of DMCs, the primary task is not simply to identify the most abundant microorganisms, but rather to recognize the keystone microbiome that truly determines fermentation direction, metabolic flux allocation, and final product quality, as well as the helper microbiome that, although not directly responsible for end-product formation, facilitates the colonization, growth, and functional expression of key members. Recent studies in fermented food microbial ecology have consistently emphasized that the stability and quality formation of traditional fermentation systems arise from complex interaction networks within microbial communities rather than from the independent action of a few dominant taxa. Accordingly, defining the “core microbiota” solely on the basis of metagenomic relative abundance may underestimate the importance of low-abundance but functionally high-leverage members [125,162]. At the same time, recent perspectives on microbial keystones suggest that key members should be identified more on the basis of their disproportionate contribution to community maintenance and system-level functional output than on their numerical dominance alone [9].

Based on this understanding, the identification of the keystone microbiome should rely on the integration of evidence from multiple biological layers. In general, metagenomics answers the questions of who is present and what functional potential these members possess, thereby providing the basis for screening candidate key taxa. Metatranscriptomics further reveals which members are truly active at a given fermentation stage, allowing discrimination between taxa that are present but inactive and those that are actively performing functions. Metaproteomics confirms whether key metabolic processes are actually being executed at the enzyme and functional protein levels. Metabolomics then links these data to organic acids, alcohols, esters, amino acid derivatives, and other flavor- or function-related metabolites, thereby establishing a continuous evidence chain from community composition to functional expression, metabolic output, and quality phenotype [33,113]. Culturomics further converts candidate members into manipulable living strains that can be used in co-culture, removal/re-addition, and simulated fermentation experiments [107,163]. In other words, the identification of keystone microbiomes should not stop at co-occurrence patterns, but should instead be based on the combined consistency of abundance, activity, function, and phenotypic contribution [125,164].

Compared with the keystone microbiome, the identification of the helper microbiome should focus more explicitly on facilitative functions. These members may not be the main executors of metabolism and may not always occur at high abundance, yet they can significantly improve the colonization ability and metabolic performance of key members through nutritional cross-feeding, substrate pretreatment, removal of inhibitory metabolites, regulation of pH and redox status, promotion of biofilm formation, or enhancement of stress tolerance [125,165,166,167]. Therefore, helper microbiomes cannot be defined solely by abundance or network centrality, but instead require cross-omics associations and experimental validation. For example, combined metagenomic and metabolomic analyses can be used to identify potential substrate-supply relationships; transcriptomic and proteomic data can reveal whether certain members activate pathways related to vitamin biosynthesis, amino acid release, or stress buffering; and co-culture or back-inoculation experiments can validate whether these members genuinely improve the growth and functional output of key microorganisms. Methodologically, the keystone microbiome answers the question of who drives the main function, whereas the helper microbiome addresses who supports the stable realization of that function [125].

Naturally fermented sourdough provides a representative case for this identification framework. Calabrese et al. [116]. combined metagenomic and metatranscriptomic analyses and found that the sourdough community was not dominated solely by a few high-abundance members, but instead comprised a hierarchically structured metabolic consortium composed of dominant, subdominant, and satellite species, each participating in different processes related to sugar metabolism, acid production, flavor formation, and community stability. Based on these multi-omics data, the researchers further reconstructed a synthetic sourdough consortium and systematically removed individual species to monitor transcriptomic changes in the community, demonstrating that certain non-dominant members, although limited in their contribution to total abundance, were critical for resilience and performance maintenance [116]. This study clearly shows that both key and helper members should be defined by their functional consequences rather than by relative abundance alone.

In the case of Baijiu, the research pathway has already advanced from identification to validation and reconstruction. In studies on Xiaoqu light-aroma Baijiu, researchers identified 10 core microorganisms by comprehensively considering abundance, flavor contribution, and co-occurrence relationships, and then constructed synthetic microbial consortia based on these findings. Modeling and fermentation validation showed that this core consortium could substitute for traditional Xiaoqu and reproduce the major fermentation process and product characteristics, demonstrating that multi-omics and related statistical integration can directly support the rational assembly of DMCs [47]. Furthermore, in a multi-metaomics study of Baobaoqu, the combined analysis of metagenomics, metatranscriptomics, and metaproteomics showed that, among the 5211 species detected by metagenomics, only 1774 were actually active. The researchers further isolated special functional microorganisms related to the formation of lactic acid, pyrazines, and phenylethanol, revealing a clear distinction between who is present and who is functioning, and further highlighting the necessity of identifying both key and helper members through multi-omics [117].

Wine research provides an especially clear demonstration of the value of identifying helper microbiomes. A 2024 multi-omics study showed that the initial yeast community composition in grape must, rather than external fermentation conditions, more strongly determined subsequent fermentation performance and the metabolic profile. In that system, dominant yeasts determined the overall fermentation outcome, whereas non-*Saccharomyces* members altered the final wine characteristics through precursor release, metabolic complementation, and environmental regulation [150]. This suggests that, in alcoholic DMCs, *Saccharomyces cerevisiae* often constitutes the keystone microbiome, whereas some non-*Saccharomyces* yeasts may be more appropriately regarded as helper microbiomes that improve fermentation performance and flavor complexity.

Therefore, in the multi-omics-guided design and optimization of DMCs, the identification of keystone and helper microbiomes should follow a basic logic: first, use multi-omics to define candidate members; then, use cultivation and reconstruction experiments to distinguish their dominant versus facilitative roles. The former determines the metabolic direction and core quality traits of the system, whereas the latter determines whether this direction can be realized efficiently, stably, and reproducibly. Only by identifying and retaining both levels can DMCs more closely approximate natural fermentation systems in terms of fermentation efficiency, community stability, and product typicity [125].

### 4.2. Assembly-Assessment-Redesign (A-A-R) Framework for DMCs Optimization

**Figure 5:**
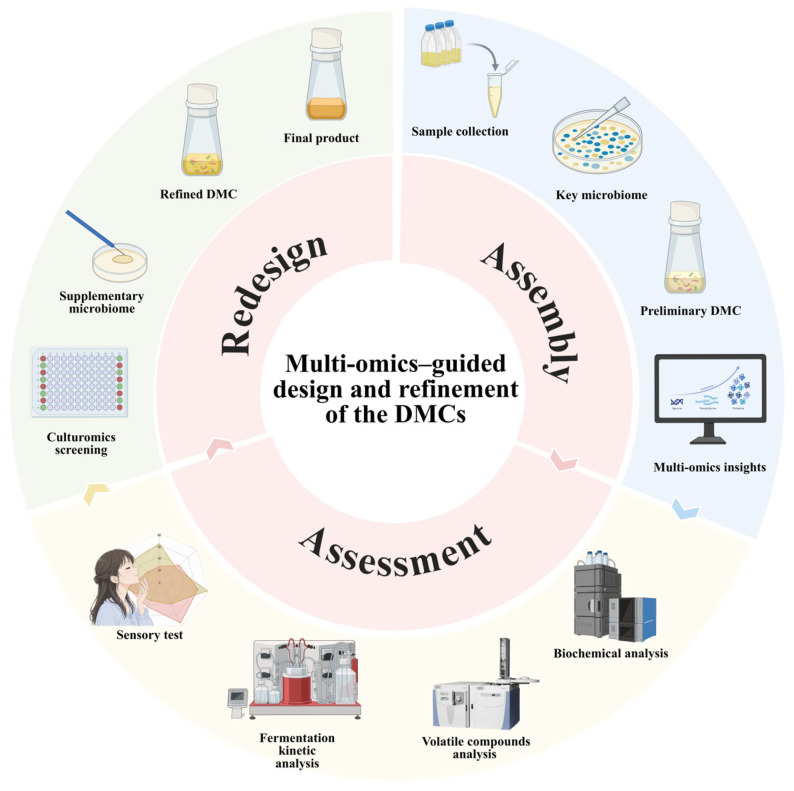
The “Assembly-Assessment-Redesign” approach of DMCs.

Although research on DMCs for traditional fermented foods has increased in recent years, most studies still remain largely at the stage of proposing or assembling preliminary consortia, whereas systematic evaluation and iterative optimization of their functional performance, community stability, and reproducibility remain relatively limited. Consequently, many initially constructed DMCs, although theoretically reasonable, may not operate stably under different fermentation conditions or fully achieve the desired flavor, quality, or functional characteristics. Therefore, to obtain efficient, stable, and predictable reconstructed fermentation systems, DMC optimization should be regarded as a continuous Assembly-Assessment-Redesign (A-A-R) process rather than a one-time microbial assemblage (Figure 5) [32,168].

(1) Assembly stage

The first step of the A-A-R framework is the assembly of candidate DMCs. The core task at this stage is to screen candidate strains from the keystone and helper microbiomes identified through previous multi-omics analyses and combine them according to functional complementarity, ecological compatibility, and process suitability. Here, “assembly” does not mean simply combining several abundant or commonly reported strains; instead, it requires comprehensive consideration of the metabolic division of labor and ecological interactions among consortium members. For example, acid production by lactic acid bacteria may create a microenvironment favorable for the growth of subsequent members, ethanol produced by yeasts may serve as a direct substrate for acetic acid bacteria, and saccharifying enzymes released by filamentous fungi may provide fermentable carbon sources for yeasts and bacteria [29]. In other words, the key objective of the Assembly stage is to establish an initial consortium structure that is functionally integrated, ecologically compatible, and practically implementable [7,8].

At this stage, the major value of multi-omics lies in rational screening. Metagenomics helps identify candidate members and their potential functions; metatranscriptomics and metaproteomics help confirm the actual activity and functional execution of these members in natural systems; metabolomics helps pinpoint key metabolic pathways and metabolites closely associated with target product quality; and culturomics further converts these candidates into manipulable isolated strains [13,42]. By integrating such information, researchers can avoid assembling DMCs solely on the basis of empirical knowledge or relative abundance, thereby improving both the rationality and the success rate of the initial design [125,169].

(2) Assessment stage

Once candidate consortia have been assembled, the second step is the systematic assessment of their fermentation performance. The purpose of the Assessment stage is not merely to determine whether fermentation can occur, but rather to comprehensively evaluate whether the DMC truly exhibits the desired target-oriented functionality [170]. Assessment indicators should generally cover four aspects. First, fermentation kinetics, including substrate consumption rate, pH change, acidity accumulation, and the formation of ethanol or other key intermediates. Second, metabolic output, including the profiles of volatile and non-volatile flavor compounds, organic acids, amino acid derivatives, and other functional metabolites. Third, community stability, including whether community composition remains stable, whether key functions are redundant, and whether significant antagonism occurs among members. Fourth, final product quality, including flavor, texture, shelf stability, and batch-to-batch reproducibility [125,168].

This stage particularly emphasizes high throughput and comparability. Recent reviews have pointed out that tools such as microplate fermentation, automated small-scale fermentation systems, and droplet microfluidics can significantly improve the efficiency of DMC assessment while helping capture subtle differences in microbial interactions and functional performance [171,172]. Through these approaches, the performance of different strain combinations, inoculation ratios, and fermentation conditions can be rapidly compared, thereby providing quantitative support for subsequent optimization. In essence, the Assessment stage uses experimental data to answer a central question: whether the currently assembled DMC is sufficiently stable, efficient, and close to the target fermentation phenotype [32,171,173].

(3) Redesign stage

If assessment results indicate that the current consortium is still inadequate in terms of fermentation efficiency, stability, flavor complexity, or batch consistency, the process should proceed to the Redesign stage. The core concept of this stage is to adjust the consortium in a targeted manner according to assessment outcomes, rather than returning to a purely empirical trial-and-error approach [8,32,171]. Common redesign strategies include adjusting the inoculation ratios of different members to strengthen specific key metabolic pathways; removing members showing strong antagonistic effects or excessive functional redundancy [6], introducing new helper strains to improve precursor supply, environmental buffering, or flavor complexity; and, where necessary, enhancing specific functions through directed breeding or strain engineering [30,174]. With the development of machine learning and computational modeling, an increasing number of studies have begun to combine multi-omics data with community dynamics prediction models to forecast the potential outcomes of different reconstruction strategies, thereby improving the efficiency and precision of Redesign [171].

From a methodological perspective, the significance of the Redesign stage lies in transforming correlative understanding into actionable optimization. In other words, multi-omics should not only be used to explain observed phenomena, but should also serve the reconfiguration of microbial consortia and the improvement of their performance. After multiple rounds of A-A-R iteration, DMCs can gradually evolve from initially feasible systems into fermentation consortia that are stable, controllable, and reproducible, ultimately approaching the standards required for industrial application.

Overall, the A-A-R framework provides a closed-loop strategy for DMC optimization, moving from rational assembly to systematic assessment and then to targeted redesign [32]. Its advantages are threefold. First, it avoids the inefficiency of repeated empirical trial and error. Second, it enables multi-omics results to be genuinely translated into engineering guidance for microbial consortium design. Third, it emphasizes that community optimization is a dynamic iterative process rather than a one-time static design [30,170]. This framework is particularly important for traditional fermented foods, because the goal is not merely to make fermentation occur, but to improve the stability, controllability, and reproducibility of the process while preserving the characteristic flavor and functional traits of traditional products. Therefore, the A-A-R framework is not only an optimization route for DMCs, but can also be regarded as an important methodological paradigm by which multi-omics drives the transition of traditional fermentation from empiricism-based production toward precision design [8,30,175].

To make the A-A-R framework more operational, each stage should be linked to clear evidence standard and a decision point. In the Assembly phase, candidate strains should not be selected only because they are abundant or repeatedly detected. Stronger evidence should include cross-omics consistency, measurable activity in the natural system, association with target metabolites, culturable availability, safety status, and ecological compatibility. A strain can enter the initial DMC only when its proposed role is supported by at least 2 types of evidence. These may include metagenomic potential, transcript or protein activity, metabolite association, and growth or interaction data from culture-based tests. Keystone members should be linked to target functions. Helper members should be linked to facilitative roles, such as precursor supply, stress buffering, pH adjustment, redox regulation, or improved colonization of keystone strains.

In the Assessment phase, success should be judged by quantitative and reproducible indicators. Fermentation kinetics should include substrate consumption, biomass growth, pH decline, acidity formation, gas release, ethanol production, or other product-specific rates. Metabolic output should include the concentration of key flavor compounds, organic acids, amino acid derivatives, alcohols, esters, safety-related metabolites, and target functional compounds. Community stability should be evaluated by the persistence of keystone and helper members, the absence of strong antagonism, and the maintenance of functional redundancy. Product performance should include sensory reproducibility, batch-to-batch consistency, and similarity to the desired traditional phenotype. These indicators should be tested across replicate batches and, where possible, across small-scale and pilot-scale systems.

Redesign should begin when one or more indicators fall outside the expected range. The adjustment should be guided by the source of failure. Slow fermentation may require a change in inoculation ratio or the addition of strains with stronger substrate conversion. Weak flavor formation may require reinforcement of precursor-generating or aroma-producing members. Poor stability may require removal of antagonistic strains or addition of helper members that improve environmental buffering. Large batch variation may require tighter control of process parameters or replacement of unstable strains. In this way, Redesign is not a return to empirical trial and error. It becomes a targeted correction based on measurable defects. After each redesign, the modified DMC should re-enter the same assessment process. This closed cycle allows DMCs to move gradually from an initially plausible community to a tested and reproducible fermentation system with defined functions, stable performance, and clearer industrial potential.

## 5. Future Prospects

Despite this progress, multi-omics-guided DMC research is still transitioning from proof of concept to broader practical application. Major challenges remain, including insufficient temporal and spatial matching among omics datasets, the lack of standardized cross-omics integration strategies, and the persistence of numerous uncultured, low-abundance, or functionally unannotated microorganisms in many fermentation systems. In addition, community structures and functional patterns identified at laboratory scale cannot always be directly extrapolated to industrial fermentation systems, which are more complex, open, and strongly affected by fluctuations in raw materials and process conditions. Accordingly, future studies should not only improve the resolution and interpretability of multi-omics analyses, but also strengthen the full-chain linkage from multi-omics-based identification to functional validation and industrial-scale application [170].

Looking ahead, deep learning and other artificial intelligence approaches are expected to become major drivers of multi-omics-guided DMC design [176,177]. Recent reviews have highlighted that AI/ML methods are particularly well suited to handling the high-dimensional, heterogeneous, and strongly nonlinear datasets generated in fermented food research, and can be used to identify key biomarkers, decipher core interaction patterns such as those between lactic acid bacteria and yeasts, and improve the accuracy of strain-feature prediction and community-function inference [178,179]. More broadly, multi-omics studies have also demonstrated the advantages of deep learning in integrating heterogeneous datasets and extracting latent association patterns, especially for cross-level analyses linking the microbiome, metabolome, and phenotype [180,181,182].

For DMC research, deep learning may be valuable in at least four respects. First, it can identify combinations of features that determine fermentation performance, moving beyond the conventional screening of single species or metabolites. Second, it can support predictive models of community dynamics, enabling early prediction of succession trajectories and functional outputs under different strain combinations, inoculation ratios, and process conditions. Third, when combined with graph-based or temporal models, it can simulate interaction strength and ecological dependencies among microbial members, thereby providing more targeted support for consortium reconstruction and redesign. Fourth, it can be integrated with explainable artificial intelligence (AI) to improve the biological interpretability of model predictions and reduce the risk of “black-box optimization” in DMC design [183,184]. Recent studies in the broader microbiome field have already demonstrated the utility of temporal machine-learning frameworks and graph neural networks for predicting community dynamics, suggesting a feasible route for transferring these approaches to DMC optimization in fermented food systems.

This trend has already begun to emerge in fermented food research. For example, a 2025 study on abnormal stacking fermentation in sauce-flavor Baijiu combined machine learning with multi-omics integration to identify microbial and flavor biomarkers associated with abnormal fermentation and to clarify key metabolic features related to quality imbalance [185]. Another study applied machine learning to global fermented food metagenomic datasets and identified a large number of previously unannotated functional enzyme clusters, while also establishing a model for classifying food origins on the basis of enzyme-cluster features [180,186]. These studies indicate that AI is no longer merely an auxiliary statistical tool, but is gradually becoming integrated into the full chain of feature discovery, mechanistic inference, fermentation warning, and design optimization.

Accordingly, future research on multi-omics-guided DMCs should advance in four major directions. First, standardized multi-omics sampling and analytical workflows with temporal and spatial matching should be established to improve comparability across studies. Second, culturomics, co-culture experiments, and simulated fermentation validation should be further strengthened to shorten the distance from association to causality. Third, deep learning, dynamic modeling, and explainable AI should be incorporated into the A-A-R framework, thereby accelerating DMC optimization from experience-driven iteration to prediction-driven iteration. Fourth, stronger integration should be built among laboratory-scale, simulated fermentation-scale, and industrial-scale validation, with the ultimate goal of developing a new generation of precision fermentation systems that combine stability, controllability, reproducibility, and traditional typicity. These future directions highlight that the development of DMCs should not only rely on omics-based association analysis, but should also move toward experimentally validated, computationally predictable, and industrially applicable fermentation design.

## 6. Conclusions

Multi-omics-guided DMC design provides a useful strategy for moving traditional fermented food research from descriptive community profiling toward functional and controllable fermentation. By integrating metagenomics, metatranscriptomics, metaproteomics, metabolomics, and culturomics, researchers can better identify functional microorganisms, clarify microbial interactions, and reconstruct consortia with improved stability, reproducibility, and target-oriented performance. The keystone/helper microbiome concept and the Assembly-Assessment-Redesign (A-A-R) framework together offer a practical logic for linking strain selection, functional validation, performance assessment, and iterative consortium improvement.

Nevertheless, this approach remains an evolving strategy rather than a fully mature solution. Its application still depends on high-quality omics datasets, cultivable strain availability, reliable functional verification, and consistency between laboratory models and industrial fermentation conditions. Future studies should therefore emphasize standardized workflows, experimental validation, cautious interpretation of association-based results, and process-scale evaluation. Artificial intelligence and machine learning may assist feature screening and predictive modeling, but their biological interpretability and transferability must be verified through microbiological experiments and fermentation trials. With stronger validation and industrial adaptation, multi-omics-guided DMCs may provide a more reliable foundation for improving fermentation stability and product quality while preserving the characteristic microbial and sensory features of traditional fermented foods.

## Figures and Tables

**Table 1:** Comparative roles of different omics approach in supporting DMC design for traditional fermented foods and beverages.

Omics Approach	Main Information Provided	Strengths	Limitations	Relevance for DMC Design
Metagenomics	Microbial composition, species or strain-level diversity, functional genes, and metabolic potential	Reveals detectable community members and potential metabolic capacity. Useful for identifying candidate core taxa, functional genes, and metabolic pathways	Cannot directly distinguish living, dead, dormant, or active cells. Functional predictions depend strongly on reference databases and annotation quality	Provides the first screening layer for candidate keystone or helper microorganisms and helps define the potential functional repertoire of a consortium
Metatranscriptomics	Actively expressed genes, transcriptional activity, and stage-specific microbial responses	Identifies metabolically active microorganisms and pathways under specific fermentation conditions. Useful for distinguishing detected taxa from active contributors	RNA is unstable and highly time-sensitive. Results are affected by sampling, rRNA depletion, sequencing depth, and reference genome quality	Prioritizes active functional members and helps select strains that respond to substrate, stress, and fermentation-stage changes
Metaproteomics	Functional proteins, enzymes, and taxa responsible for biochemical execution	Provides more direct evidence of functional execution than DNA- or RNA-based methods. Useful for identifying enzymes involved in substrate degradation and flavor formation	Protein extraction from fermented matrices is difficult. Low-abundance proteins may be missed, and database dependence can limit protein identification	Validates whether candidate strains actually perform key metabolic tasks and supports the clarification of functional division of labor within DMCs
Metabolomics	Volatile and non-volatile metabolites, flavor compounds, nutritional components, and safety-related compounds	Directly reflects terminal metabolic phenotypes and product quality attributes. Useful for linking microbial activity with sensory, nutritional, and safety outcomes	Cannot independently identify which microorganisms produce or regulate specific metabolites. Annotation of unknown metabolites remains challenging	Defines target phenotypes for DMC reconstruction and provides quality-oriented criteria for evaluating consortium performance
Culturomics	Living, isolable, and manipulable strains from complex communities	Converts omics discoveries into available microbial resources. Enables strain preservation, co-culture experiments, and functional validation	Captures only the cultivable fraction of the community and is affected by medium, temperature, oxygen, and incubation conditions	Provides the strain resources required for assembling, testing, and redesigning DMCs in controlled fermentation systems

## Data Availability

No new data were created or analyzed in this study. Data sharing is not applicable to this article.
